# Evaluation of Short Term and Long Term Subjective and Objective Cognitive Outcomes Following ECT for Depression in a Naturalistic Ambulatory Setting: A Cohort Study

**DOI:** 10.31083/AP45286

**Published:** 2026-02-03

**Authors:** Sarah A. Goegan, Gary M. Hasey, Jelena P. King, Bruno J. Losier, Peter J. Bieling, Margaret C. McKinnon, Heather E. McNeely

**Affiliations:** ^1^Department of Psychology, Neuroscience & Behaviour, McMaster University, Hamilton, ON L8S 4L8, Canada; ^2^Clinical Neuropsychology Services, St. Joseph's Healthcare Hamilton, Hamilton, ON L8N 3K7, Canada; ^3^Trauma and Recovery Research Unit, Homewood Research Institute, Guelph, ON N1E 6K9, Canada; ^4^Department of Psychiatry & Behavioural Neurosciences, McMaster University, Hamilton, ON L8N 3K7, Canada; ^5^ECT Clinic, St. Joseph's Healthcare Hamilton, Hamilton, ON L8N 3K7, Canada; ^6^Mood Disorders Treatment and Research Clinic, St. Joseph's Healthcare Hamilton, Hamilton, ON L8N 3K7, Canada; ^7^Schizophrenia and Community Integration Service, St. Joseph's Healthcare Hamilton, Hamilton, ON L8N 3K7, Canada

**Keywords:** cognitive outcomes, naturalistic study, electroconvulsive therapy (ECT), major depression, subjective memory

## Abstract

**Background:**

This cohort study examined changes in cognitive outcomes, subjective memory, and depressive symptoms in an understudied area: electroconvulsive therapy (ECT) delivered in a naturalistic ambulatory setting with a heterogeneous, clinically complex sample of individuals with mixed mood disorders.

**Methods:**

Participants were adults (mean age = 45.7 years; female gender = 69%) receiving ambulatory ECT for a major depressive episode (Major Depressive Disorder = 81.4%; Bipolar Spectrum Disorder = 18.9%); 62.9% had at least 1 co-occurring mental health diagnosis. Clinical and cognitive assessments were completed at baseline (n = 100), mid-ECT (n = 94), 2–4 weeks (n = 64), 6-months (n = 34), and 12-months (n = 19) post-ECT. Neurocognitive performance was assessed using the Repeatable Battery for Assessment of Neuropsychological Status® (RBANS) at all timepoints, except mid-ECT and subjective memory was assessed using the Squire Subjective Memory Questionnaire (SSMQ).

**Results:**

Overall, cognitive performance was lower than expected compared to premorbid estimates at baseline but did not significantly worsen following ECT (*p* > 0.05), with the exception of a transient decline in verbal fluency scores. Patients endorsed elevated subjective memory complaints before and after ECT, which differed by treatment response as indicated by a significant Time by Response Group interaction *p* = 0.039. There were significant main effects of time in both ‘Responders' (≥50% improvement in Beck Depression Inventory [BDI-II] score post-ECT), *p* < 0.001 and ‘Non-Responders' (<50% improvement in BDI-II) *p* = 0.021. Within group, after controlling for multiple comparisons, there was a clear trend for SSMQ scores to improve across most time points in the ‘Responder' group, but subjective memory declined and remained around baseline level in the ‘Non-Responder' group across follow-up. In the sample as a whole, rapid reduction in BDI-II scores from baseline to mid-ECT predicted rapid improvement in SSMQ scores, *p* = 0.013.

**Conclusions:**

Clinically complex adults referred to ECT for depression presented with prominent memory concerns and performed below expectation compared to their estimated premorbid cognitive functioning at baseline. Naturalistic delivery of ECT did not appear to be associated with prolonged adverse cognitive outcomes; however, subjective memory concerns and below-expected cognitive performance persisted during follow-up. Treatment response impacted subjective memory outcomes, with only ‘Responders' endorsing slightly reduced, though still persistent, subjective memory concerns following ECT. Conclusions on the long-term impacts of ECT are tempered by the high lost to follow up (LTFU) rate observed across follow-up assessments (66% LTFU at 6-months, 81% LTFU at 12-months). Nonetheless, these findings emphasize the need to address subtle cognitive deficits and memory complaints that persist following ECT, even in individuals demonstrating clinical improvement.

## Main Points

(1) Adults presenting for electroconvulsive therapy (ECT) for a major depressive episode in a naturalistic setting evidenced a high occurrence of psychiatric co-morbidities, with anxiety disorders being most common.

(2) ECT delivered to a clinically complex cohort under naturalistic conditions was not associated with persistent objective cognitive declines, although there was a transient worsening of verbal fluency during the course of treatment.

(3) Despite absence of persistent objective cognitive decline, patients endorsed persistent subjective cognitive concerns. This persistent concern was evident even among those who responded to ECT although some relative improvement in subjective cognitive concern was associated with improved mood.

(4) In addition to measuring depressive symptoms, screening for co-occurring mental health conditions such as anxiety disorders is recommended, as unresolved co-morbidities may contribute to persistent subjective cognitive concerns and worse outcomes.

(5) Addition of adjunctive psychosocial interventions including psychoeducation, cognitive remediation and cognitive behaviour therapy (CBT) may be beneficial to address residual subjective memory concerns and improve outcomes in this clinical population.

## 1. Introduction

Electroconvulsive therapy (ECT) is a highly efficacious treatment for severe unipolar depression [1, 2, 3] and bipolar depression [4]. Despite demonstrated efficacy, care providers and patients are often reluctant to prescribe or to undergo ECT [5, 6]. This reluctance is due, in part, to concerns around adverse cognitive side effects, particularly a worsening of memory [7, 8, 9].

Indeed, a recent meta-analysis demonstrated a decrease in autobiographical memory, verbal fluency, and verbal memory in the short-term (i.e., 1 to 28 days) following ECT with small to medium effect sizes [10]. Retrograde and anterograde amnesia symptoms immediately following ECT are also commonly reported [7, 11]. Fortunately, cognitive impairment appears mostly transient, with performance returning to baseline levels about 1month post-ECT [3, 10, 12, 13]. This finding appears to be the case for many areas of cognition, including verbal and visual episodic memory, verbal fluency, and executive functioning [10, 12, 13]. Further, there is growing evidence that performance in certain cognitive domains may improve significantly after the acute treatment phase of ECT [10, 12, 14]. For instance, Mohn and Rund [15] found significant improvements in processing speed, attention, and visual learning 6-weeks post-ECT compared to baseline. However, recent synthesis research suggests some adverse cognitive impacts, particularly in learning capabilities, may persist long-term [16]. Notably, ECT parameters (e.g., electrode placement, number of treatment sessions) can differentially impact the clinical and cognitive effects of ECT [14].

It is important to note here that the cognitive impact of ECT is confounded by the cognitive symptoms of depression. Cognitive deficits, such as reduced processing speed, attention, immediate memory, and executive functioning, are often present in individuals with depression [17, 18] and may partly, although not entirely, remit as mood improves [19]. Thus, remission of mood symptoms following ECT may partially explain the observed improvements in cognitive functioning [7, 14].

As expected, depressed patients often report significant subjective cognitive complaints before receiving ECT [20] and a worsening of subjective memory functioning attributed to ECT [7, 11, 21, 22, 23]. To highlight this, a recent meta-analysis reported a weighted mean prevalence rate of 48.1% for patients reporting subjective cognitive complaints, with memory difficulties the most commonly reported [22].

Notably, subjective assessment of cognition and actual performance on objective neuropsychological testing may be poorly correlated [7, 15, 21, 24]. Subjective memory is often closely related to mood state, with more subjective memory complaints being associated with greater severity of depressive symptoms [7, 20, 25]. For instance, individuals in an acute depressive episode appear prone to greater underestimation of cognitive abilities, when considering their objective cognitive performance on attention and memory measures [24]. Additionally, improvements in subjective memory after ECT have been associated with improvement in depressive symptom severity [26]. However, other factors, such as aspects of meta-cognition (i.e., awareness of one’s own thoughts) and executive functioning, may also help explain the discrepancy between subjective and objective cognition [24].

There is a paucity of research examining the subjective and objective cognitive impact of ECT administered under naturalistic conditions. Here, naturalistic conditions are defined as less restrictive inclusion and exclusion criteria for the provision of ECT, particularly regarding complex psychiatric co-morbidities, and adjustments to ECT parameters made with clinical discretion rather than according to fixed protocol. Under these naturalistic conditions, in some instances, ECT may be offered as a last resort to many who are extremely treatment-resistant or are acutely suicidal, despite comorbidities or adverse psychosocial circumstances that would otherwise be considered poor prognostic indicators. Certainly, the provision of ECT outside of controlled research studies involves a more heterogeneous collection of patients and greater variability in treatment parameters compared to those often reported in the extant literature. To examine the impact this heterogeneity has on outcomes, our ECT research team conducted a naturalistic study of ECT for depression in a cohort of ambulatory adults with mixed mood disorders and co-morbidities referred for ECT over a ten-year period. Details on the sample characterization and main clinical findings are reviewed in depth in a previous publication [27].

The cognitive effects of ECT have mainly been explored in studies employing relatively restrictive inclusion/exclusion criteria. The cognitive changes seen in the more heterogeneous population treated with ECT in the naturalistic setting have not been as extensively examined. Greater knowledge of the short- and long-term effects of naturalistic ECT on both subjective and objective cognitive functioning will inform more ecologically valid clinical discussions around the risks and benefits of ECT in naturalistic patient populations.

The objectives of the current study were to examine the short- and long-term impact of naturalistically delivered ECT on cognitive functioning, including immediate and delayed memory, attention, visuospatial abilities, processing speed, and working memory, and subjective memory appraisals in a clinically heterogeneous cohort. Additional objectives were to explore the relationships between subjective memory, cognitive performance, and clinical symptomatology. Lastly, we aimed to examine whether improvement in depressive symptoms predicted changes, if present, in cognitive functioning and subjective memory. Other objectives are explored in separate reports, such as rates of treatment response and remission, changes in severity of depressive symptoms and functioning, and predictors of treatment response and functional outcomes [27].

We predicted that, at baseline, subjective memory would not be associated with objective memory, but would be significantly associated with depression severity, and would be inversely related to clinical response to ECT. Further, we hypothesized that cognitive functioning would not significantly worsen when tested 2 to 4 weeks post-ECT or at 6- and 12-month follow-up assessments. We predicted that, if present, cognitive improvements would be found in areas known to be impacted by depressive symptoms, including processing speed, attention, and executive functioning, and that improvement in depressive symptoms would significantly predict these changes in cognitive performance. Further, we expected to observe subjective memory appraisals significantly worsen during ECT and then significantly improve following ECT (i.e., post-ECT and long-term follow-up) compared to baseline levels. Finally, we expected that changes in depressive symptoms would predict changes in subjective memory.

## 2. Materials and Methods

### 2.1 Study Design and Setting

Data was collected using a pre-post ECT cohort design with longitudinal follow-up assessment points of 2 to 4 weeks post-ECT, 6 months, and 12 months post-ECT. The study received ethics approval from the Research Ethics Board of St. Joseph’s Healthcare Hamilton (SJHH) and the Hamilton Integrated Research Ethics Board (ID 11052). As approved, the pre-ECT and 2-to 4-week post-ECT assessments were delivered as part of standard clinical care. The 6-month and 12-month follow-up assessments were not part of standard clinical care and participants received compensation of $ 40 per assessment and paid hospital parking.

**Fig. 1.**
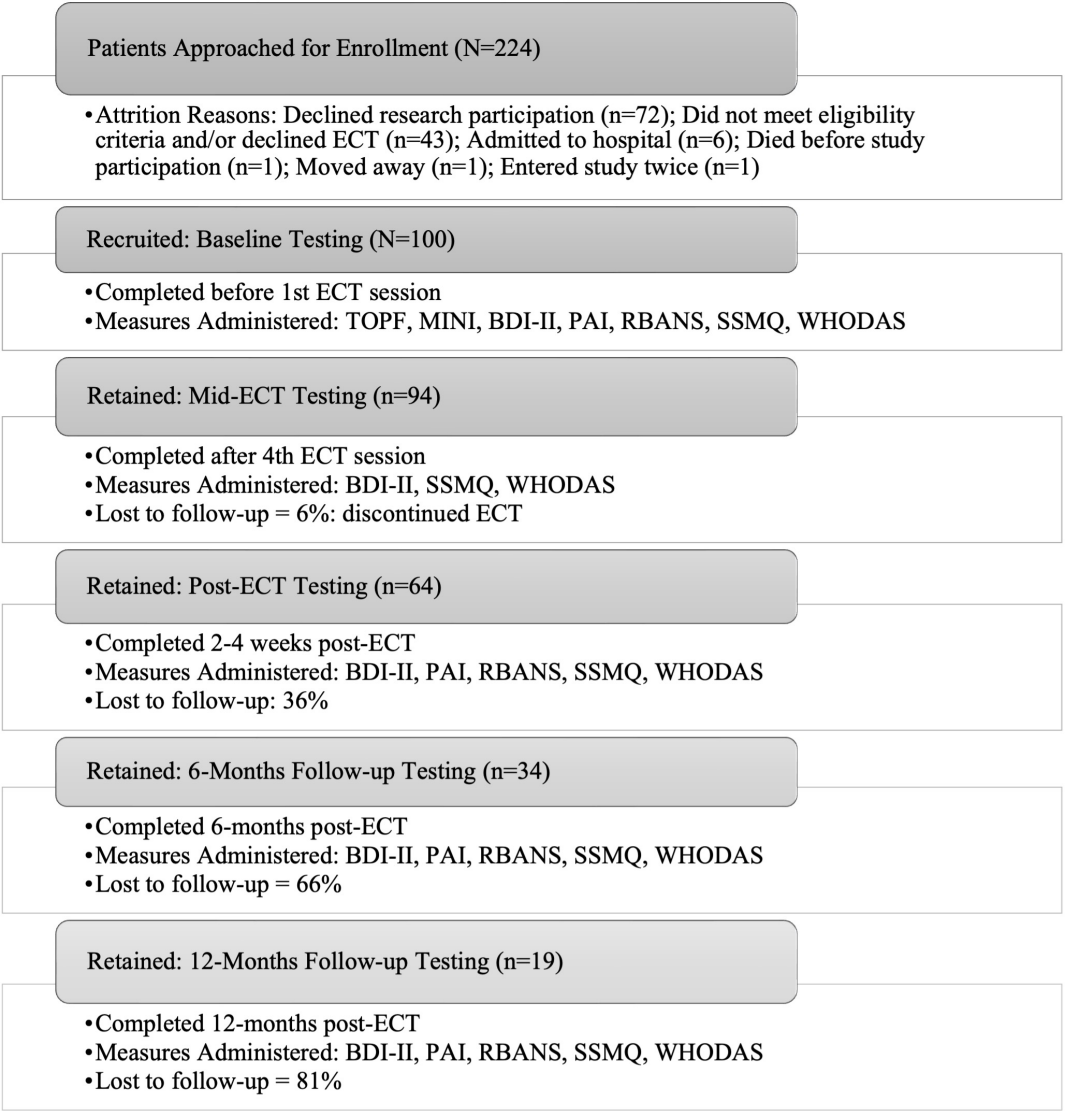
**Study flow diagram**. Illustration of number patients approached, number and percent recruited and retained in the study protocol or lost to follow up (LTFU), timing of testing sessions over 12 months and tests administered at each time point. Abbreviations: ECT, electroconvulsive therapy; TOPF, Test of Premorbid Function; MINI, Mini International Neuropsychiatric Interview; BDI-II, Beck Depression Inventory-II; PAI, Personality Inventory; RBANS, Repeatable Battery for the Assessment of Neuropsychological Status; SSMQ, Squire Subjective Memory Questionnaire; WHODAS, World Health Organization Disability Assessment Schedule.

Participants were recruited from a naturalistic sample of adults receiving an acute course of ECT between September 2010 and November 2020 at the ambulatory ECT clinic at SJHH an urban academic healthcare centre affiliated with McMaster University and the second largest provider of mental health services in Ontario Canada with a large urban, suburban, and rural catchment area. Clinical criteria for referral for ECT for depression included: inadequate response to at least three prior adequate trials of antidepressant medication, or acutely suicidal; and for bipolar depression in addition to three failed antidepressant medication trials at least one adequate trial of a mood stabilizer; or acutely suicidal. Inclusion criteria: referred to SJHH ambulatory ECT clinic for treatment of unipolar or bipolar depressive episode, age 18 to 75 years, any gender, normal or corrected to normal vision, ability to comprehend and communicate in English, medically cleared for ECT treatment. Exclusion criteria: diagnosis of Major Neurocognitive Disorder (dementia), primary psychotic disorder, Intellectual Disability, Acquired Brain Injury with loss of consciousness, visually impaired or unable to communicate adequately in English for testing purposes. Patients were evaluated by an ECT psychiatrist and anesthesiologist to determine suitability for ECT before being approached to participate in this study. Eligible patients were informed about the study by the ECT nurse, and those who expressed interest were contacted by telephone by the neuropsychology administrative assistant and scheduled for their baseline assessment where they provided written informed consent administered by a research team member. They were contacted by neuropsychology staff by telephone to arrange all assessment visits. Assessments were performed pre-ECT, mid-ECT, within 2 to 4 weeks post-ECT, at 6-months and 12-months post-ECT in an ambulatory hospital setting in the Clinical Neuropsychology Services or ECT Clinic of SJHH by a licensed clinical neuropsychologist and/or clinical or research staff under the direct supervision of a licensed clinical neuropsychologist. The administrative staff randomized patients on recruitment to receive test order A or B at baseline and alternate versions of selected neuropsychological tests were administered at subsequent assessment visits (see Fig. 1 for additional information on assessments across timepoints).

ECT was administered twice weekly with a Thymatron^TM^ System IV ECT Instrument (40182 and 42922, Somatics, LLC, Venice, FL, USA) using bilateral temporal/bitemporal (BIL) or right unilateral (RUL) electrode placement [28] determined based on discussion between the patient and ECT physician. Most participants (82%) received BIL temporal electrode placement, 6% received RUL, 6% started BIL and switched to RUL, and another 6% started RUL and switched to BIL based on clinical decision making. Patients received either brief pulse or ultra-brief pulse ECT. The ECT psychiatrist clinically monitored response to ECT. Typical number of sessions of ECT delivered in a course of naturalistic ECT in this setting is 12. Mean number of treatments administered in this cohort was 11.1 (range = 2 to 19) with those receiving fewer sessions either showing significant response resulting in completion termination of the series or electively withdrawing from ECT before completing treatment. The length of the ECT series and adjustments to the treatment plan (e.g., electrode placement, stimulus energy, frequency), were determined by the ECT team in consultation with the ECT recipient and family. The patient’s regular outpatient treatment team were informed of the patient’s progress through the course of ECT and advised of any recommended medication changes via the weekly ECT team progress note (see [27] for more details).

### 2.2 Measures

Advanced Clinical Solutions, Test of Premorbid Functioning (TOPF) [29]. The TOPF is a single word reading test developed to predict premorbid general intellectual ability in adults. Participants read aloud a list of words that increase in complexity and familiarity until a discontinue criterion is reached. Scale scores (SS) with a mean of 100 and standard deviation of 10 are calculated.

Beck Depression Inventory-II (BDI-II) [30]. The BDI-II is a 21-item self-report measure used to assess severity of depressive symptoms. Participants are asked to rate each item reflecting on their experience over the preceding two weeks, with item ratings from 0 (none) to 3 (severe). Total score is calculated with severity ratings as follows: 0–13 = minimal, 14–19 = mild, 20–28 = moderate, 29–63 = severe.

MINI International Neuropsychiatric Interview (MINI) version 7.0.0 [31, 32]. The MINI is a clinician administered interview developed and validated as a brief structured interview for the major Axis I psychiatric disorders in the Diagnostic and Statistical Manual of Mental Disorders 4th Edition (DSM-IV) and International Classification of Diseases, 10th Revision (ICD-10).

The Personality Assessment Inventory (PAI) [33, 34]. The PAI is a 344-item self-report inventory used to measure psychological and emotional constructs and symptoms associated with DSM-IV diagnostic classifications and personality features. Each item is rated on a 4-point scale from ‘not at all true’ to ‘very true’. The PAI yields 22 non-overlapping scales, including validity scales, clinical scales, treatment scales, and interpersonal scales. The full length PAI was completed by participants at baseline and the 160-item short form PAI (PAI-SF) was administered at all post-ECT follow up visits. The PAI and PAI-SF yield 11 clinical scale scores: Somatic Complaints (SOM), Anxiety (ANX), Anxiety-Related Disorders (ARD), Depression (DEP), Mania (MAN), Paranoia (PAR), Schizophrenia (SCZ), Borderline Features (BOR), Antisocial Features (ANT), Alcohol Problems (ALC) and Drug Problems (DRG).

Repeatable Battery for Assessment of Neuropsychological Status® (RBANS) [35]. The RBANS consists of 12 subtests and was used to measure objective cognitive functioning in five domains: Immediate memory (List Learning, Story Memory), Delayed memory (List Recall, List Recognition, Story Recall, Figure Recall), Visuospatial Constructional (Figure Copy, Line Orientation), Language (Picture Naming, Semantic Fluency), and Attention (Digit Span, Coding). To minimize practice effects, the standardized equivalent alternate forms of the RBANS were used in repeat assessment (Form A and Form B). RBANS variables of interest included the Immediate Memory, Delayed Memory, and Visuospatial Constructional indices, the Coding subtest (measure of processing speed), Digit Span subtest (measure of working memory), and the Semantic Fluency subtest (measure of semantic retrieval and executive functioning). Standardized scores (SS; mean = 100, SD = 10) were calculated with higher scores reflecting better cognitive performance. The RBANS was selected for its clinical utility, the availability of normative data, alternate test versions, and ease of administration.

Squire Subjective Memory Questionnaire (SSMQ) [36]. The SSMQ is an 18-item self-report measure originally developed to assess memory before and after ECT. Participants are asked to think about how they were functioning “compared to before I began to feel bad and went to the hospital” and rate a variety of statements on a nine-point scale ranging from ‘worse than ever before’ (–4) to ‘better than ever before’ (+4). A total score (range: –72 to +72) is computed by summing the item responses, with negative scores indicating a subjective worsening of memory functioning.

The World Health Organization’s Disability Assessment Schedule 2.0 (WHODAS 2.0) [37]. The WHODAS 2.0 is a 36-item self-report measure developed to assess health and disability functioning in daily life across 6 dimensions: Cognition, Mobility, Self-care, Getting along with others, Life activities, Participation. Higher total scores reflect greater disability (score range: 0–48).

### 2.3 Statistical Methods

Statistical analyses were conducted using IBM® SPSS® Statistics Version 27 (IBM Corp., Armonk, NY, USA) with a 0.05 alpha level, two-tailed. Descriptive statistics (mean, standard deviation, percentages) were used to describe the sample demographics and characteristics. Due to the naturalistic design of the study, data is missing from various measures across time points as indicated. Missing data were excluded using pairwise deletion on an analysis-by-analysis basis and test assumptions were met unless specified within individual analyses. Data imputation methods were not used given the extent of missing data. Baseline associations between SSMQ and cognitive performance (RBANS SS) with other relevant clinical variables were explored using Pearson correlations (rp) or Spearman’s rho (rs) correlations for non-normally distributed variables (Shapiro-Wilk *p* < 0.05)

In linear mixed model analyses, the selected covariance type and estimation model were selected based on a combination of study design (e.g., number of timepoints) and Akaike Information Criterion (AIC)/Bayesian Information Criterion (BIC) optimization. Normality of residuals was evaluated using Shapiro-Wilk test. Treatment response and effectiveness of naturalistic ECT at reducing depressive symptoms was evaluated using linear mixed modelling. For statistical analyses in the current study, post-ECT Remitters and Responders were collapsed into one group, named Responders, and compared to Non-Responders (See Table 1). A five (Time) × two (Response Group) linear mixed model for SSMQ scores using AR(1) Heterogenous repeated covariance type and maximum likelihood (ML) estimation was conducted to evaluate change in subjective cognition over time; however, the model residuals were not normally distributed (Shapiro-Wilk = 0.971, *p* < 0.001). The linear mixed model was run excluding three outliers (±3 SD) on the SSMQ, which then passed the normality assumption (Shapiro-Wilk = 0.971, *p* = 0.052). Four (Time) × two (Group) linear mixed models using AR(1) Heterogenous repeated covariance type and maximum likelihood estimation were conducted for each of the RBANS SS variables of interest. When there was a significant interaction, simple main effects were conducted across Time within each response Group. When model residuals were not normally distributed, non-parametric Friedman’s Two-Way ANOVA by Ranks were performed. To examine whether post-ECT performance on the RBANS remained significantly lower than expected when compared to baseline TOPF estimated premorbid cognition, a series of paired-samples t-tests or Wilcoxon Signed Ranks tests for non-normally distributed variables (Delayed Memory, Digit Span, and Coding) were performed. Post-hoc analyses were Bonferroni corrected for multiple comparisons using a corrected alpha threshold of 0.05 divided by the number of comparisons in each analysis as specified in relevant results sections. For correlational analyses, Cohen’s guidelines for Pearson’s r and Spearman rho (rs) = 0.10, 0.30, and 0.50 are used to interpret observed effect sizes as small, medium, and large, respectively. Cohen’s d observed effect sizes of d = 0.2, 0.5, and 0.8 are interpreted as small, medium, and large, respectively. Bonferroni corrections were not applied to correlational analyses.

**Table 1.** **Baseline descriptive statistics by post-ECT response group**.

		Responders (n = 27)		Non-Responders (n = 37)	
Baseline characteristic		M (SD)	Valid n	M (SD) & Valid n	
Age (yrs)		44.5 (12.3)	26	47.5 (8.3)	37
Education (yrs)		15.2 (3.0)	26	14.2 (2.4)	37
Num sessions in 1st AC		11.7 (2.4)	26	11.4 (3.5)	37
TOPF		108.4 (11.9)	23	107.7 (10.4)	35
RBANS					
	Immediate Memory	98.3 (13.5)	25	97.9 (17.1)	37
	Delayed Memory	98.2 (10.8)	25	96.6 (15.8)	37
	Visuospatial	99.7 (17.4)	25	100.0 (16.9)	37
	Digit Span	102.6 (13.1)	26	102.5 (16.8)	37
	Coding	93.6 (16.5)	26	96.6 (17.1)	37
	Fluency	99.7 (11.8)	26	95.8 (14.3)	37
SSMQ		–19.4 (28.4)	26	–25.3 (19.6)	36
BDI-II		36.5 (8.6)	26	42.6 (8.1)	37
WHODAS		21.8 (9.8)	24	26.1 (9.6)	35
Personality assessment inventory					
	DEP	82.1 (11.9)	25	90.5 (10.5)	33
	ANX	66.4 (14.4)	25	74.1 (11.8)	33
	SOM	65.2 (12.2)	25	68.2 (11.7)	33
	BOR	63.4 (9.8)	25	66.1 (12.3)	33
	STR	58.0 (12.0)	24	57.6 (10.7)	33
	SUI	73.1 (20.6)	25	81.7 (21.2)	33
	ARD-T	54.9 (12.0)	25	69.6 (16.8)	33
		% of valid n	Valid n	% of valid n	Valid n
Sex (female/male)		69.2/30.8	26	73.0/27.0	37
Currently employed		42.1	19	24.1	29
Hx of past ECT		35.0	20	31.0	29
100% BIL ECT (Yes)		69.2	26	78.4	37
#of AC (1/>1)		84.6/15.4	26	67.6/32.4	37
Maintenance ECT (Yes)		26.9	26	32.4	37
MINI diagnoses					
	MDD	79.2	24	80.6	36
	Bipolar	20.8	24	19.4	36
	≥2 diagnoses	44.0	25	73.0	37

# refers to number. AC, Acute ECT course(s); BIL, Bitemporal electrode placement MDD, Major Depressive Disorder; RBANS indices and subtests reported are standardized scores. Personality Assessment Inventory scales are reported in T-scores and include: DEP, Depression; ANX, Anxiety; SOM, Somatic Complaints; BOR, Borderline Features; STR, Stress; SUI, Suicidal Ideation; ARD-T, Anxiety-Related Disorders-Traumatic Stress subscale.

Absolute change scores were computed for variables that showed a significant effect of Time (i.e., SSMQ, RBANS Fluency SS, and Immediate Memory SS) by subtracting baseline scores from 2 to 4 week post-ECT scores. To examine whether changes in depressive symptoms predicted changes in subjective and/or cognitive functioning across time, a series of linear mixed-effects models were conducted using AR(1) Heterogenous repeated covariance type and restricted maximum likelihood (REML) estimation. We examined the effects of time (five levels) and ΔBDI change scores (continuous, person-centered change in BDI-II score from baseline) on scores on the SSMQ and RBANS SS variables of interest. To further explore whether changes in depressive symptoms predicted changes in subjective memory and cognitive performance, additional absolute change scores were computed for mid-ECT and 2 to 4 week-post-ECT BDI-II scores (Mid-ECT minus Baseline and Post-ECT minus Baseline, respectively) and mid-ECT SSMQ scores (Mid-ECT minus Baseline). Mid-ECT and post-ECT SSMQ change scores were non-normally distributed (Shapiro-Wilks *p* < 0.05). Spearman rho (rs) correlations were conducted for these variables, showing similar results to the linear regressions; thus, only the linear regression findings are reported.

To examine whether patient’s primary diagnosis (MDD versus Bipolar Disorder) and/or presence versus absence of clinical comorbidities was associated with post-ECT changes in outcome variables of interest, a series of independent-samples *t*-tests or Mann-Whitney U tests for non-normally distributed variables (SSMQ, Delayed Memory, and Coding) were performed. Variables of interest included post-ECT change scores on SSMQ, BDI-II, or RBANS SS Immediate Memory, Delayed Memory, Visuospatial Construction, Semantic Fluency, Digit Span, and Coding.

## 3. Results

### 3.1 Participants

Eligible patients (n = 224) were approached and 100 met inclusion and exclusion criteria, consented to research, and were enrolled in the cohort (M = 45.7 years old, SD = 11.0, 69% women). The sample had a mean level of education just above post-secondary (M = 14.4 years, SD = 2.5). At baseline, all of the cohort met criteria on the MINI for a current or past Major Depressive Episode and/or had baseline BDI-II score in the severe range (BDI-II; M = 41.0, SD = 9.4, n = 99). Primary MINI diagnoses (n = 97), were Major Depressive Disorder (n = 79; 81.4%) and bipolar spectrum disorders (n = 18; 18.9%). At least one MINI diagnostic comorbidity was identified for 62.9% of the sample with the majority being anxiety disorders (see 27 for further details). Mean TOPF estimated premorbid intellectual ability registered in the average range compared to standardized normative data. Baseline cognitive performance on the RBANS was also in the average range, but was significantly lower than expected compared to the mean TOPF SS [27].

The number of participants completing evaluations and percentage lost to follow up (LTFU) at various points is shown in Fig. 1. Participants were LTFU due to being admitted to hospital and discontinuing ECT (n = 6), 1 moved away, 1 died of other causes, and the remainder declined follow-up testing, did not respond to scheduling telephone calls, or could not be scheduled for 6- and/or 12-month visits due to coronavirus disease of 2019 (COVID-19) related research restrictions.

### 3.2 Treatment Response

Treatment response and effectiveness of naturalistic ECT at reducing depressive symptoms in this heterogeneous cohort is reviewed thoroughly in a previous publication [27]. Briefly, we found that for the group as a whole, BDI-II score improved significantly by the 4th ECT session (*p* < 0.001) and this was maintained at 2 to 4 weeks (*p* < 0.001), 6-months (*p* < 0.001), and 12-month post-ECT assessments (*p* = 0.003) [27]. At 2 to 4 weeks post-ECT (n = 64), the clinical remission rate was found to be 27% (Remitters: BDI-II score ≤13 and ≥50% improvement from baseline, n = 17), with a treatment response rate of 42.2% (≥50% improvement in BDI-II score from baseline; n = 27, including remitters). Non-Responders sample (<50% improvement in BDI-II score) made up 58.7% (n = 37) of the cohort who completed the 2 to 4-week post-ECT assessment. These findings should be interpreted with caution in the context of a 36% 2 to-4-weeks post-ECT attrition rate. Differences between those who completed post-ECT testing and those LTFU were explored previously [27]. Briefly, those LTFU had significantly lower scores at baseline on the RBANS Immediate Memory index (*p* = 0.022) and the Delayed Memory Index (*p* < 0.001), and were less likely to have received previous ECT (*p* = 0.005), compared to those who completed post-ECT testing.

### 3.3 Association Between Subjective and Objective Cognition at Baseline 

As shown in Table 2, SSMQ score was not significantly correlated with RBANS SS on Immediate Memory, Delayed Memory, Visuospatial Constructional, Digit Span, Coding, or Fluency. SSMQ score was weakly correlated with RBANS Visuospatial Constructional index (rs = 0.20, *p* = 0.050) and trended towards correlation with Digit Span subtest score (rs = 0.1, *p* = 0.067).

**Table 2.** **Spearman’s rho (rs) correlations between baseline SSMQ and baseline RBANS variables**.

Variable	*n*	*M*	*SD*	SSMQ (*rs*)
SSMQ	97	–25.7	21.1	–
Immediate Memory	98	95.1	15.7	–0.13
				*p* = 0.207
Delayed Memory	98	93.0	15.4	–0.08
				*p* = –0.466
Visuospatial	98	98.0	16.1	0.20
				*p* = 0.050
Digit Span	100	101.3	15.9	–0.19
				*p* = 0.067
Coding	100	93.3	16.5	–0.03
				*p* = 0.784
Fluency	100	96.7	15.4	–0.03
				*p* = 0.774

RBANS indices and subtests reported are standardized scores. Cohen’s guidelines for Pearson’s r and Spearman rho (rs) = 0.10, 0.30, and 0.50 are used to interpret observed effect sizes as small, medium, and large, respectively.

### 3.4 Association Between Cognitive and Clinical Variables at Baseline

As shown in Table 3, SSMQ score was significantly negatively correlated with PAI DEP (rs = –0.24, *p* = 0.021), STR (rs = –0.23, *p* = –0.032), and SUI (rs = –0.2, *p* = 0.049) scales, such that worse subjective memory appraisals were associated with higher levels of self-reported depression, stress, and suicidality symptoms. Delayed Memory scores were negatively correlated with ANX (rs = –0.2, *p* = 0.013) and BOR Features (r = –0.32, *p* = 0.002) scales, and Visuospatial Constructional scores were significantly negatively correlated with the ANX (r = –0.27, *p* = 0.009) and SOM (rs = –0.27, *p* = 0.008) scales, such that higher clinical symptomatology was associated with worse cognitive performance. There were no significant correlations between any clinical variables and RBANS Immediate Memory, Digit Span, Coding, and Fluency.

**Table 3.** **Pearson correlations or Spearman rho (rs) correlations between clinical variables and subjective and objective cognition at baseline**.

Variable	*M*	*SD*	SSMQ (*rs*)	RBANS	RBANS	RBANS	RBANS	RBANS	RBANS
				Immediate Memory	Delayed Memory (*rs*)	Visuospatial	Digit Span	Coding	Fluency (*rs*)
BDI-II	41.0	9.4	–0.20	–0.09	–0.12	–0.003	–0.11	–0.05	–0.06
			*p* = 0.056	*p* = 0.378	*p* = 0.255	*p* = 0.975	*p* = 0.265	*p* = 0.644	*p* = 0.579
			SD = 97	n = 97	n = 97	n = 97	n = 99	n = 97	n = 99
WHODAS	24.7	10.0	–0.17	–0.17	–0.14	–0.14	–0.07	–0.04	–0.11
			*p* = 0.102	*p* = 0.109	*p* = 0.196	*p* = 0.193	*p* = 0.479	*p* = 0.687	*p* = 0.291
			n= 91	n = 93	n = 93	n = 93	n = 93	n = 93	n = 93
DEP (*rs*)	88.0	11.1	–0.24	–0.01	–0.15	–0.07	–0.10	0.06	–0.04
			*p* = 0.021	*p* = 0.892	*p* = 0.148	*p* = 0.513	*p* = 0.370	*p* = 0.574	*p* = 0.711
			n = 91	n = 92	n = 92	n = 92	n = 92	n = 92	n = 92
ANX	71.1	12.7	–0.17	–0.14	–0.26	–0.27	–0.08	0.01	–0.02
			*p* = 0.100	*p* = 0.183	*p* = 0.013	*p* = 0.009	*p* = 0.459	*p* = 0.903	*p* = 0.832
			n = 91	n = 92	n = 92	n = 92	n = 92	n = 92	n = 92
SOM (*rs*)	67.5	11.6	–0.16	–0.06	–0.14	–0.27	–0.03	–0.04	–0.03
			*p* = 0.132	*p* = 0.548	*p* = 0.190	*p* = 0.008	*p* = 0.780	*p* = 0.697	*p* = 0.807
			n = 91	n = 92	n = 92	n = 92	n = 92	n = 92	n = 92
BOR	66.5	11.6	–0.15	–0.13	–0.32	–0.16	–0.06	–0.02	0.07
			*p* = 0.158	*p* = 0.224	*p* = 0.002	*p* = 0.139	*p* = 0.566	*p* = 0.874	*p* = 0.497
			n = 91	n = 92	n = 92	n = 92	n = 92	n = 92	n = 92
STR	59.1	11.2	–0.23	–0.08	–0.16	–0.15	–0.04	0.06	0.02
			*p* = 0.032	*p* = 0.436	*p* = 0.128	*p* = 0.162	*p* = 0.718	*p* = 0.602	*p* = 0.874
			*n* = 87	*n* = 88	*n* = 88	n = 88	n = 88	n = 88	n = 88
SUI (*rs*)	80.2	22.5	–0.21	–0.02	–0.05	0.08	–0.14	0.12	0.15
			*p* = 0.049	*p* = 0.838	*p* = 0.620	*p* = 0.443	*p* = 0.187	*p* = 0.244	*p* = 0.165
			*n* = 91	*n* = 92	*n* = 92	n = 92	n = 92	n = 92	n = 92
ARD-T (*rs*)	66.2	17.8	–0.10	–0.002	–0.16	–0.10	–0.17	–0.03	–0.004
			*p* = 0.372	*p* = 0.988	*p* = 0.129	*p* = 0.344	*p* = 0.122	*p* = 0.783	*p* = 0.974
			n = 91	n = 92	n = 92	n = 92	n = 92	n = 92	n = 92

RBANS, Repeatable Battery for the Assessment of Neuropsychological Status indices and subtests reported are standardized scores (Immediate Memory, Delayed Memory, Visuospatial, Digit Span, Coding, Fluency). Personality Assessment Inventory scales are reported in T-scores and include: DEP, Depression; ANX, Anxiety; SOM, Somatic Complaints; BOR, Borderline Features; STR, Stress; SUI, Suicidal Ideation; ARD-T, Anxiety-Related Disorders-Traumatic Stress subscale. Cohen’s guidelines for Pearson’s r and Spearman rho (rs) = 0.10, 0.30, and 0.50 are used to interpret observed effect sizes as small, medium, and large, respectively.

### 3.5 Change in Subjective Memory and Cognitive Performance 

**Fig. 2.**
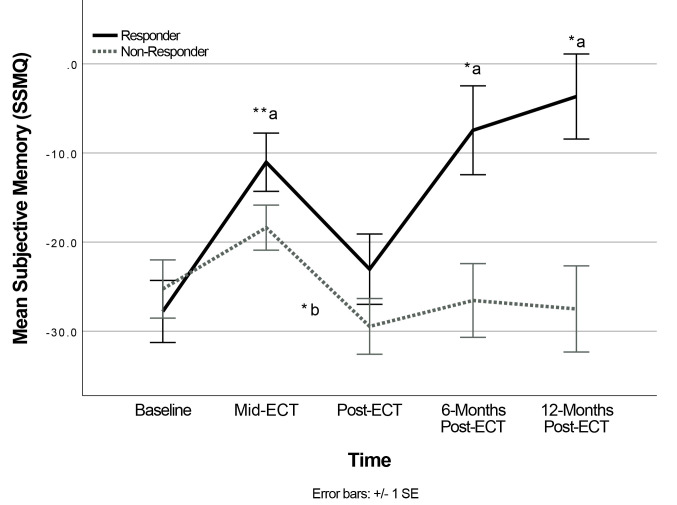
**Change in Subjective Memory following ECT (Time × Group)**. Change in subjective memory scores on the Squire Subjective Memory Questionnaire (SSMQ) across Time and Response Groups (Responders and Non-Responders). Baseline n = 97 Mid-ECT n = 92, Post-ECT n = 59, 6-months Post-ECT n = 30, 12-months Post-ECT n = 15. *p < 0.05, **p < 0.01. Post-hoc analyses for Responder group are marked with ‘_a_’ and comparisons are made from Baseline to identified timepoint. Post-hoc analyses with Non-Responder group are marked ‘_b_’ and comparison is made from Mid-ECT to Post-ECT. Error bars reflect standard error of the mean.

For subjective memory, there was a significant interaction of Time × Group, *F*(4,46.06) = 2.75, *p* = 0.039. A significant main effect of Time was found in the Responder group, *F*(4,24.57) = 6.97, *p* < 0.001, and the Non-Responder group, *F*(4,40.02) = 3.25, *p* = 0.021, but post-hoc analyses revealed a different pattern of change for each treatment response group (see Fig. 2).

Responders’ SSMQ scores significantly improved from baseline to mid-ECT (*p* = 0.009), returned to levels comparable to baseline at post-ECT (*p* = 1.00), then significantly improved by 6-months (*p* = 0.034), and 12-months post-ECT (*p* = 0.015) compared to baseline. In contrast, the Non-Responder group mean SSMQ significantly worsened from mid-ECT to post-ECT (*p* = 0.020) and did not show significant differences between baseline and any subsequent time points (*p* > 0.05). Notably, these post-hoc analyses were no longer significant after Bonferroni correction for multiple comparisons (corrected *p* < 0.005).

**Fig. 3.**
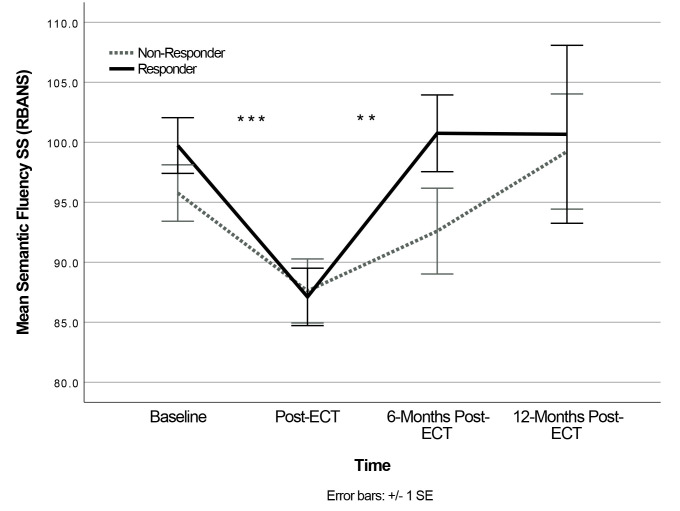
**Change in semantic fluency following ECT (Time × Group)**. Change in RBANS Semantic Fluency standardized scores (SS) across Time and Response Groups (Responders and Non-Responders). Baseline n = 99, Post-ECT n = 64, 6-months Post-ECT n = 34, 12-months Post-ECT n = 19. **p < 0.01, ***p < 0.001 for post-hoc analysis for Time collapsed across Group. Error bars reflect standard error of the mean.

Main effects and interaction effects for Immediate Memory, Digit Span, and Coding were non-significant (*p* > 0.05). There was no significant effect of Time (*p* > 0.05), for either Delayed Memory or Visual Constructional indices. The Semantic Fluency model showed a significant main effect of Time, *F*(3,36.17) = 10.25, *p* < 0.001, and non-significant effects for Group and Time × Group interaction. Post-hoc analyses (Bonferroni corrected *p* < 0.008) revealed that Semantic Fluency scores significantly worsened from baseline to post-ECT (*p* < 0.001; Cohen’s d = 0.55), and improved significantly from post-ECT to 6-months post-ECT (*p* = 0.007; Cohen’s d= 0.43) (see Fig. 3).

Interestingly, removing Group from the model, revealed a significant main effect of Time for RBANS Immediate Memory, *F*(3,42.42) = 3.76, *p* = 0.018. Post-hoc analyses showed that Immediate Memory scores significantly improved from baseline (n = 99) to 2-to-4-week (n = 64) post-ECT (*p* = 0.017), but baseline scores did not differ significantly from 6-month (n = 34) or 12-month (n = 19) follow-up. At 2 to 4 week post-ECT testing (n = 64), RBANS SS performance again fell in the average descriptor range (SS: 90–110) for all indices and subtests of interest, except for Semantic Fluency, which fell in the low average range. Post-ECT RBANS SS (n = 64) for Delayed Memory (M = 99.13, SD = 15.71), z = –3.34, *p* < 0.001, Coding (M = 95.24, SD = 15.75), z = –4.31, *p* < 0.001, and Semantic Fluency (M = 87.79, SD = 14.44), *t*(57) = 9.88, *p* < 0.001, d = 1.297 remained significantly lower than expected compared to estimated premorbid function per baseline TOPF SS (M = 107.98, SD = 10.91). Post-ECT RBANS SS for Digit Span (Wilcoxon Signed Ranks, *p* = 0.032), Immediate Memory (*p* = 0.030), and Visuospatial Construction (*p* = 0.056) were not significantly different from TOPF SS after Bonferroni correction.

### 3.6 Association Between Changes in Subjective Memory and Changes in Cognitive Performance

There were no significant correlations between baseline-to-post-ECT change in SSMQ and change in any of the RBANS change variables (*p* > 0.05) (see Table 4).

**Table 4.** **Spearman rho (rs) correlations between baseline-to-post-ECT absolute change in SSMQ and RBANS scores**.

Variable	n	M	SD	SSMQ change
SSMQ change	60	–3.9	29.3	–
Immediate Memory change	57	4.5	15.0	0.11
				*p* = 0.423
Fluency change	60	–9.9	16.3	0.15
				*p* = 0.263

Absolute change scores were computed by subtracting Baseline scores from 2–4 weeks post-ECT scores. For change in RBANS indices and subtests, standardized scores were used.

### 3.7 Predicting Change in Subjective Memory and Cognitive Performance

#### 3.7.1 Depressive Symptoms

There were significant main effects for both Time (*F*(4,41.97) = 3.16, *p* = 0.024) and ΔBDI (*F*(1,56.43) = 13.04, *p* < 0.001) on SSMQ score. The Time × ΔBDI interaction was non-significant, *p* > 0.05. The Visuospatial Constructional and Semantic Fluency models both showed significant main effects of Time, *F*(3,38.31) = 4.29, *p* = 0.011 and *F*(3,34.76) = 4.04, *p* = 0.014, respectively, and non-significant effects for ΔBDI and Time × ΔBDI interaction; however, the residuals for both models were not normally distributed. Main effects and interaction effects were non-significant for Immediate Memory, Delayed Memory, Digit Span, and Coding. Post-hoc analyses for the main effects of Time were not conducted as these were explored in earlier analyses.

Mid-ECT BDI-II change scores significantly predicted mid-ECT SSMQ change scores, *F*(1,89) = 6.36, *p* = 0.013, *R*^2^ = 0.067, such that greater rapid reduction in depressive symptoms predicted greater rapid improvement in subjective memory appraisal. However, baseline to post-ECT change in BDI-II scores did not significantly predict post-ECT change scores for SSMQ or RBANS Fluency and Immediate Memory indices, *p* > 0.05.

#### 3.7.2 Psychiatric Diagnoses

There were no significant differences between primary diagnoses of MDD and Bipolar Disorder on any of the variables of interest, Bonferroni corrected *p* > 0.006. Likewise, there were no significant differences between those with the presence or absence of clinical comorbidities (as determined by the MINI) on any of the variables of interest, *p* > 0.006.

#### 3.7.3 ECT Duration

The total number of acute ECT sessions received was not significantly correlated with post-ECT absolute change scores for SSMQ (rho = –0.063, *p* = 0.635), RBANS Fluency index (rho = –0.074, *p* = 0.573), or Immediate Memory index (rho = 0.007, *p* = 0.957). Unfortunately, we were unable to statistically examine the extent to which other ECT parameters predicted subjective and objective cognitive performance due to insufficient sample variability.

## 4. Discussion

This study followed a cohort of clinically heterogeneous, severely depressed adults with a high proportion of co-morbid psychiatric diagnoses receiving ECT under naturalistic conditions in an ambulatory mental health setting to examine the relations between, and changes in, cognitive functioning, subjective memory appraisals, and clinical mental health symptomatology before and shortly after receiving ECT and at 6- and 12-month follow-up.

Before undergoing ECT, the sample as a whole was severely depressed, most with a diagnosis of Major Depressive Disorder (MDD) and just under 20% with a diagnosis of a Bipolar Spectrum Disorder (BD). Almost 63% of the cohort met diagnostic criteria for 2 or more psychiatric diagnoses, and all reported substantial subjective memory concerns. As discussed in an earlier publication [27], clinical response and remission rates were lower than expected in comparison to controlled clinical trials and slightly lower than that seen in other more naturalistic patient samples. This may be related to the higher rates of psychiatric co-morbidities in the present sample. Participants without psychiatric comorbidities demonstrated better post-ECT response (58.3%) and remission (45.8%) rates, versus lower response (29%) and remission (13.2%) rates in patients with at least one psychiatric comorbidity. See [27] for further discussion of response rates and sample characteristics in comparison to the extant literature.

In keeping with what would be expected in the context of high baseline burden of depression and anxiety symptoms associated with negative cognitive bias and high functional impairment, on entry to the study, all participants reported subjective memory concerns with highest subjective memory impairment being associated with more severe symptoms of depression, stress and suicidality. However, subjective memory appraisals were not significantly correlated with any of the objective cognitive domains measured, including processing speed, working memory, immediate memory, delayed memory, visuospatial construction, and semantic fluency which all registered within normative limits albeit significantly below expectation compared to premorbid estimates of this sample. One may expect normatively intact cognitive performance to be associated with fewer subjective memory complaints; however, the lack of statistical association between subjective and objective memory performance is consistent with the lack of concordance often reported in the literature, where subjective concerns tend to be more consistently associated with mood state [15, 21, 38, 39]. One previous study found that patients in the acute phase of a major depressive episode with severe depressive symptoms showed a significant association between higher subjective cognitive complaints and worse objective cognition performance [24], and in addition, these patients also underestimated their objective cognitive performance, and the underestimation was predicted by higher depressive symptoms and better performance on executive function tests [24]. The authors suggested that the relation between higher executive function and greater underestimation of cognitive abilities may suggest better awareness of subtle cognitive weaknesses not detected on standard objective tests [24] and our findings of a statistically significant discrepancy between premorbid general intellectual ability and normatively intact but lower baseline cognitive performance supports this hypothesis that patients may be aware of subtle declines. However, others have proposed that self-reported memory complaints are more reflective of a general negative self-evaluation style, common in mood disorders [38]. We also found that before starting ECT, worse subjective memory was significantly correlated with higher ratings of depression symptoms as well as higher endorsement of stress and suicidality. These findings are in line with previous research that demonstrates strong connections between mood state and subjective memory that have been consistently found in both depressed and control samples [15, 21, 24, 40]. It is also important to consider the impact of a potential “recall-anchor” bias in subjective memory ratings using the SSMQ as this measure prompts participants to rate their current subjective memory against their pre-illness perceived functioning. Given that most patients presenting for ECT for depression have lived with unresolved depression and associated impairment for many years, their subjective comparison of their current state to their pre-depression level of functioning is likely suspect based on both normal age-related changes that may not be taken into account in addition to the negative self-appraisal common in depression. In contrast, objective cognitive testing is compared against age-controlled normative data, and thus is not subject to this potential bias. Together, the current results suggest a complex interplay between awareness of subtle cognitive changes that may be compounded by recall bias and negative bias attributable to severe clinical symptomatology in this patient population.

Cognitive impairment is a common feature across diverse psychiatric illnesses and has been associated with symptom severity [41, 42]. Interestingly, in the current cohort, depressive symptoms, as measured by the BDI-II and PAI, were not significantly correlated with any of the objective cognitive domains assessed. However, greater severity of other mental health symptoms including anxiety, borderline personality features and somatic concerns were associated with worse objective cognitive performance on entry to the cohort prior to initiation of ECT. These results suggest that among naturalistic samples receiving ECT, greater clinical complexity reflected by other mental health symptoms in addition to depression may be relevant contributors to subjective and objective cognitive outcomes and should be assessed and considered.

### 4.1 Changes in Subjective Memory Following ECT

All patients reported subjective memory concerns prior to ECT and continued to perceive their memory as worse than it was before the onset of their depressive disorder even among those whose depression responded to ECT, possibly due to some degree of recall bias and/or a persistent general negative cognitive bias associated with low mood. Importantly, subjective memory concerns did change significantly over the course of treatment. Although results of post-hoc analyses did not remain significant after statistical correction for multiple comparisons, there was a trend for subjective memory complaints to vary with treatment response. For Responders, subjective memory appraisals improved rapidly from baseline to mid-ECT in association with a rapid improvement in mood, returned to baseline at post-ECT, then improved by the 6-month and 12-month assessments. In contrast, Non-Responders’ subjective memory appraisals worsened from mid-ECT to post-ECT, with no significant improvements from baseline to any subsequent timepoints. In the sample as a whole, although rapid reduction in depressive symptoms at mid-ECT significantly predicted early improvements in subjective memory appraisals, changes in depressive symptoms from baseline to 2 to 4-weeks post-ECT did not predict changes in subjective memory during the same period. These findings are generally consistent with literature showing improvements in subjective cognition from pre- to post-ECT that is moderated by improvements in depressive symptoms consistent with the effects of mood congruent negative cognitive bias [22].

As mentioned above, it is also important to consider the nature of the subjective memory measure, the SSMQ, and its implications for interpreting the post-ECT subjective memory findings. The SSMQ requires a respondent to consider their memory functioning “compared to before I began to feel bad and went to the hospital” with options ranging from ‘worse than ever before’ (–4) to ‘better than ever before’ (+4). This requires accessing remote autobiographical memories, which are often impaired in depressed individuals who tend to experience an overgeneralization or lack of specificity when recalling autobiographical events [43, 44]. In the case of chronic depression lasting many years, this may also be confounded by cognitive decline associated with aging. Further, appraisals of current memory functioning in depressed individuals are thought to be influenced by a negative self-evaluation style. Thus, it is plausible that we are seeing an over-estimation of past abilities and under-estimation of current abilities in this clinical sample, and that this bias persists following ECT treatment. Another limitation when interpreting these findings is the high proportion of our sample that was lost to follow-up.

Despite the caveats to interpreting subjective memory scores, our findings suggest that substantial significant subjective memory complaints were present before and after ECT, even for those who saw a significant reduction or remission of depressive symptoms. Notably, these subjective memory complaints occurred in the context of stable or in some areas improved objective cognitive performance indicating that patients believed their memory was impaired even when their measured cognition had improved or registered at normative levels for their age. Beliefs about memory may continue to influence self-perception of functional capacity and mood, which then further impacts on daily memory abilities. Indeed, persistent subjective memory complaints are not uncommon following ECT [9, 22, 23]. A recent meta-analysis reported a weighted mean prevalence rate of 48.1% of patients reporting subjective cognitive complaints following ECT [22] and a systematic review on patients’ perspectives of ECT noted that rates of persistent subjective memory loss post-ECT ranged from 26% to 55% [9, 23]. Importantly, the finding that patients who responded to ECT still experienced persistent subjective memory complaints highlights the importance of evaluating subjective cognition as relevant outcomes of ECT in tandem to evaluating reduction in depressive symptoms and objective cognition.

### 4.2 Changes in Cognitive Functioning Following ECT

Encouragingly, objective cognitive performance generally did not worsen following ECT, except for transient worsening in semantic fluency that returned to baseline levels by 6-months post-ECT. Cognitive performance on the RBANS remained largely unchanged following naturalistic ECT, and immediate memory showed significant improvement from baseline to the 2 to 4 weeks post-ECT. In line with these findings, a meta-analysis of ECT practices suggested that verbal fluency, along with verbal memory and autobiographical memory, tend to show a transient worsening in the short-term (i.e., 1 to 28 days) with return-to-baseline or improvement in the long-term (i.e., >1 month) post-ECT [10]. Another recent meta-analysis found long-term adverse effects of ECT on learning capacity, with long-term improvements in executive function and processing speed, and stability in most other cognitive domains [16].

In the current study, changes in subjective memory from baseline to 2 to 4 weeks post-ECT were not correlated with changes in performance on immediate memory or semantic fluency measures. Similarly, changes in depressive symptoms from baseline to 2 to 4 weeks post-ECT did not predict changes in any of the cognitive domains assessed during the same period. Although overall cognitive performance did not appear to worsen, it is important to recognize that before beginning ECT our patient cohort as a whole was functioning at a level slightly lower than expected compared to their estimated premorbid intellectual functioning. At post-ECT, although not significantly worse and within normative limits, participants were still performing below expectation on measures of delayed memory, working memory/executive function (coding) and semantic fluency compared to estimated premorbid score suggesting subtle persistent declines in cognition compared to premorbid functioning. Indeed, cognitive impairment is not uncommon in depressed samples [41], and has been found to persist into euthymic periods [45, 46], particularly in patients with a greater illness burden (e.g., higher number of previous episodes, longer illness duration) [47] and may be mediated by reduction in hippocampal volume [48, 49]. These volume changes are reportedly correlated with working memory/executive functioning [49]. Although ECT has been associated with increase in hippocampal volume [50], the persistent relative impairment observed in our sample could be a consequence of the pathophysiology of depression.

Importantly, another consideration is that the RBANS may not be sufficiently sensitive to capture nuanced changes in specific domains of cognitive functioning. The memory tests employed in this study looked primarily at anterograde memory, namely the ability to form new memories before and after ECT. However, studies looking at the types of memory loss reported by ECT patients indicates that impairments to retrograde memory, particularly that of autobiographical memory, may be of greater concern [9]. Although the current study did not include a measure of autobiographical memory, the test battery did include a semantic fluency task that involved searching one’s semantic memory stores for information that matches a category cue and quickly retrieving that information, assessing elements of semantic memory and executive functioning. Whereas immediate and delayed memory tasks assess the ability to encode and retrieve new information, semantic memory relies on the ability to retrieve previously learned decontextualized knowledge and this process did transiently decline during ECT.

Interestingly, both semantic memory and executive control have been implicated in autobiographical memory functioning in healthy and depressed samples [51, 52, 53, 54]. As noted, post-ECT reductions in verbal fluency (an element of semantic memory) and autobiographical memory following ECT are fairly common [10, 38]. Fortunately, impairments in verbal fluency and autobiographical memory appear to resolve fairly quickly post-ECT [10, 38]. Thus, our finding of a transient worsening of semantic fluency post-ECT that resolved by 6-months follow-up appears in line with this literature. However, patients’ subjective reports of amnesia seem to be more persistent, lasting beyond six months post-ECT [38]. It will be important for future research to examine factors contributing to persistent subjective memory loss and the potential tertiary outcomes associated with these difficulties, such as functional impairment, perceptions of ECT and risk of depression relapse. In the interim, clinicians are encouraged to be proactive in discussing and normalizing subjective memory concerns with this patient population. Psychoeducation about the various factors that influence subjective memory appraisals within a depression population, including negative cognitive bias and recall bias, should be discussed. Clinicians are also encouraged to utilize the research findings to support patients to re-appraise subjective concerns using a Cognitive Behaviour Therapy (CBT) approach. For example, using a CBT approach, clinicians may guide clients to challenge their assumptions or appraisals of poor memory (i.e., subjective impairment) against the evidence (i.e., lack of objective memory impairment in research samples, or possibly in their own cognitive test results if available) which may help patients reframe their causal attributions and gain a more balanced and less biased evaluation of their current status, which anecdotally often leads to improved mood and subjective memory outcomes. Providing patients with more extensive psychoeducation related to cognitive biases and subjective memory challenges associated with depression that are distinguished from objective challenges may also aid in shifting beliefs about the negative impact of ECT on memory and may eventually contribute to reduced colloquial stereotypes about ECT.

## 5. Limitations and Future Directions

Conclusions on the long-term cognitive impacts of naturalistic ECT are tempered by the high LTFU rate observed across follow-up assessments (66% LTFU at 6-months, 81% LTFU at 12-months) which weaken statistical power and hamper generalizability of the results. Given the long duration of follow-up in this study, one must consider potential issues associated with selection bias. Post-ECT, a high proportion of participants did not return calls to schedule follow-up visits or actively declined to continue with repeat assessment. Administrative staff recorded many files as “closed” without explicitly documenting the reason. It is possible that those who continued to experience symptoms of depression or subjective cognitive impairment may have been more motivated to remain engaged in follow-up, while those who had better treatment response or higher remission rates may have returned to pre-morbid levels of social and occupational functioning and as such were less motivated or available for repeat assessment visits. This potential selection bias could help explain lower than expected overall response and remission rates. This could be addressed in future studies by including more detailed reasons for attrition rates, by including virtual testing to decrease burden associated with travel time to participate in follow up, and/or by using brief online or telephone symptom screening for all participants including those who decline to participate in more fulsome clinical and cognitive follow-up. Unfortunately, medication data and some ECT treatment data (e.g., seizure duration, seizure threshold, type of anesthetic used) was not included in the research database, and as such we are unable to determine the extent to which differences in these factors may have impacted response rates and cognitive outcomes. That being said, given this was a naturalistic sample and none of these factors would have been controlled, statistically evaluating the discrete impact of these factors in this sample would remain challenging. Future research in naturalistic settings should nevertheless include medication and other treatment data, as well as additional clinical data points such as age of onset and illness duration, in order to better understand the nuanced relationships between psychosocial, individual and treatment factors as these impact functional and clinical outcomes.

## 6. Conclusions

In a cohort of adult patients with unipolar and bipolar depression and psychiatric co-morbidities referred for ambulatory ECT for depression, naturalistically delivered ECT was not associated with cognitive declines, although there was a transient worsening in semantic fluency, and immediate memory showed significant improvement at 2 to 4 weeks post-ECT. Improvement in depressive symptoms following ECT did not predict gains in objective cognitive functioning post-ECT but was associated with subjective memory improvements. Worse subjective memory appraisals were significantly correlated with more severe depressive symptoms at baseline, as well as with higher self-reported stress and suicidality. In addition, more severe levels of anxiety, BOR , and SOM were associated with worse delayed memory and/or visuospatial constructional performance before ECT. Critically, persistent subjective memory complaints post-ECT and at extended follow-up evaluations, as well as subtle but significant relative cognitive declines compared to premorbid estimates, were evident across the sample, even in those whose depression responded to ECT. Further research is needed to understand the factors contributing to persistent subjective memory concerns, as well as how we may best address these concerns for our patients. Researchers and clinicians may consider the possible benefit of adjunctive psychoeducation related to the impact of depression, anxiety and stress on cognition and related to the probable transient negative impact of ECT on cognitive functioning during the course of treatment. Cognitive remediation therapies that specifically target cognitive dysfunction in mood disorder populations may also represent a beneficial adjunctive treatment [55]. Adjunctive cognitive behavioural therapies (CBT) may help to challenge negative biased thinking characteristic of depression which may be contributing to increased subjective memory concerns through over-focusing on normative cognitive slips, and assessment and treatment of co-occurring mental health symptoms such as generalized anxiety would be warranted as these symptoms may be over-looked and remain unresolved in the context of ECT for depression but may continue to negatively impact both mood and subjective cognition. Prior to implementing this naturalistic study, ECT physicians at the host institution were not routinely measuring co-occurring mental health symptoms and cognition. Implementation of measurement-based care in ambulatory ECT clinics to address all co-occurring mental health and cognitive symptoms beyond depression alone may be critical in tailoring follow-up care and relapse prevention appropriately and may improve outcomes in clinically complex and heterogeneous samples.

## Availability of Data and Materials

Due to the clinically sensitive nature of the research and the conditions of our ethics approval, supporting data cannot be made openly available.

## References

[1] Kho KH, van Vreeswijk MF, Simpson S, Zwinderman AH (2003). A meta-analysis of electroconvulsive therapy efficacy in depression. *The Journal of ECT*.

[2] Pagnin D, de Queiroz V, Pini S, Cassano GB (2008). Efficacy of ECT in depression: a meta-analytic review. *Focus*.

[3] UK ECT Review Group (2003). Efficacy and safety of electroconvulsive therapy in depressive disorders: a systematic review and meta-analysis. *Lancet (London, England)*.

[4] Dierckx B, Heijnen WT, van den Broek WW, Birkenhäger TK (2012). Efficacy of electroconvulsive therapy in bipolar versus unipolar major depression: a meta-analysis. *Bipolar Disorders*.

[5] Byrne P, Cassidy B, Higgins P (2006). Knowledge and attitudes toward electroconvulsive therapy among health care professionals and students. *The Journal of ECT*.

[6] McFarquhar TF, Thompson J (2008). Knowledge and attitudes regarding electroconvulsive therapy among medical students and the general public. *The Journal of ECT*.

[7] American Psychiatric Association (2008). The practice of electroconvulsive therapy: recommendations for treatment, training, and privileging (A task force report of the American Psychiatric Association).

[8] Kellner CH, Greenberg RM, Murrough JW, Bryson EO, Briggs MC, Pasculli RM (2012). ECT in treatment-resistant depression. *The American Journal of Psychiatry*.

[9] Rose D, Fleischmann P, Wykes T, Leese M, Bindman J (2003). Patients’ perspectives on electroconvulsive therapy: systematic review. *BMJ (Clinical Research Ed.)*.

[10] Landry M, Moreno A, Patry S, Potvin S, Lemasson M (2021). Current Practices of Electroconvulsive Therapy in Mental Disorders: A Systematic Review and Meta-Analysis of Short and Long-Term Cognitive Effects. *The Journal of ECT*.

[11] Enns MW, Reiss JP (1992). Electroconvulsive therapy. *The Canadian Journal of Psychiatry*.

[12] Semkovska M, McLoughlin DM (2010). Objective cognitive performance associated with electroconvulsive therapy for depression: a systematic review and meta-analysis. *Biological Psychiatry*.

[13] Vasavada MM, Leaver AM, Njau S, Joshi SH, Ercoli L, Hellemann G (2017). Short- and Long-term Cognitive Outcomes in Patients With Major Depression Treated With Electroconvulsive Therapy. *The Journal of ECT*.

[14] Stippl A, Kirkgöze FN, Bajbouj M, Grimm S (2020). Differential Effects of Electroconvulsive Therapy in the Treatment of Major Depressive Disorder. *Neuropsychobiology*.

[15] Mohn C, Rund BR (2016). Significantly improved neurocognitive function in major depressive disorders 6 weeks after ECT. *Journal of Affective Disorders*.

[16] Guo Q, Wang Y, Guo L, Li X, Ma X, He X (2024). Long-term cognitive effects of electroconvulsive therapy in major depressive disorder: A systematic review and meta-analysis. *Psychiatry Research*.

[17] Castaneda AE, Tuulio-Henriksson A, Marttunen M, Suvisaari J, Lönnqvist J (2008). A review on cognitive impairments in depressive and anxiety disorders with a focus on young adults. *Journal of Affective Disorders*.

[18] Lee RSC, Hermens DF, Porter MA, Redoblado-Hodge MA (2012). A meta-analysis of cognitive deficits in first-episode Major Depressive Disorder. *Journal of Affective Disorders*.

[19] Gonda X, Pompili M, Serafini G, Carvalho AF, Rihmer Z, Dome P (2015). The role of cognitive dysfunction in the symptoms and remission from depression. *Annals of General Psychiatry*.

[20] Mohn C, Rund BR (2016). Neurocognitive profile in major depressive disorders: relationship to symptom level and subjective memory complaints. *BMC Psychiatry*.

[21] Prudic J, Peyser S, Sackeim HA (2000). Subjective memory complaints: a review of patient self-assessment of memory after electroconvulsive therapy. *The Journal of ECT*.

[22] Semkovska M, Knittle H, Leahy J, Rasmussen JR (2023). Subjective cognitive complaints and subjective cognition following electroconvulsive therapy for depression: A systematic review and meta-analysis. *The Australian and New Zealand Journal of Psychiatry*.

[23] Brus O, Nordanskog P, Båve U, Cao Y, Hammar Å, Landén M (2017). Subjective Memory Immediately Following Electroconvulsive Therapy. *The Journal of ECT*.

[24] Serra-Blasco M, Torres IJ, Vicent-Gil M, Goldberg X, Navarra-Ventura G, Aguilar E (2019). Discrepancy between objective and subjective cognition in major depressive disorder. *European Neuropsychopharmacology: the Journal of the European College of Neuropsychopharmacology*.

[25] Coleman EA, Sackeim HA, Prudic J, Devanand DP, McElhiney MC, Moody BJ (1996). Subjective memory complaints prior to and following electroconvulsive therapy. *Biological Psychiatry*.

[26] Vann Jones S, McCollum R (2019). Subjective memory complaints after electroconvulsive therapy: systematic review. *BJPsych Bulletin*.

[27] Goegan SA, Hasey GM, King JP, Losier BJ, Bieling PJ, McKinnon MC (2022). Naturalistic Study on the Effects of Electroconvulsive Therapy (ECT) on Depressive Symptoms. *Canadian Journal of Psychiatry. Revue Canadienne De Psychiatrie*.

[28] Swartz CM, Nelson AI (2005). Rational electroconvulsive therapy electrode placement. *Psychiatry (Edgmont (Pa.: Township))*.

[29] Wechsler D (2009). Advanced clinical solutions for the WAIS-IV and WMS-IV.

[30] Beck AT, Steer RA, Brown G (1996). Beck depression inventory–II. *Psychological Assessment*.

[31] Lecrubier Y, Sheehan DV, Weiller E, Amorim P, Bonora I, Sheehan KH (1997). The Mini International Neuropsychiatric Interview (MINI). A short diagnostic structured interview: reliability and validity according to the CIDI. *European Psychiatry*.

[32] Sheehan DV, Lecrubier Y, Sheehan KH, Amorim P, Janavs J, Weiller E (1998). The Mini-International Neuropsychiatric Interview (M.I.N.I.): the development and validation of a structured diagnostic psychiatric interview for DSM-IV and ICD-10. *The Journal of Clinical Psychiatry*.

[33] Morey LC (1996). An interpretive guide to the Personality Assessment Inventory (PAI). *Psychological Assessment Resources*.

[34] Morey LC (2007). Personality assessment inventory (PAI): Professional manual.

[35] Randolph C, Tierney MC, Mohr E, Chase TN (1998). The Repeatable Battery for the Assessment of Neuropsychological Status (RBANS): preliminary clinical validity. *Journal of Clinical and Experimental Neuropsychology*.

[36] Squire LR, Wetzel CD, Slater PC (1979). Memory complaint after electroconvulsive therapy: assessment with a new self-rating instrument. *Biological Psychiatry*.

[37] Üstün TB, Kostanjsek N, Chatterji S, Rehm J (2010). Measuring health and disability: Manual for WHO disability assessment schedule WHODAS 2.0.

[38] Fraser LM, O’Carroll RE, Ebmeier KP (2008). The effect of electroconvulsive therapy on autobiographical memory: a systematic review. *The Journal of ECT*.

[39] Srisurapanont M, Suttajit S, Eurviriyanukul K, Varnado P (2017). Discrepancy between objective and subjective cognition in adults with major depressive disorder. *Scientific Reports*.

[40] Schweizer S, Kievit RA, Emery T, Henson RN (2018). Symptoms of depression in a large healthy population cohort are related to subjective memory complaints and memory performance in negative contexts. *Psychological Medicine*.

[41] Bowie CR, Gupta M, Holshausen K (2013). Cognitive remediation therapy for mood disorders: rationale, early evidence, and future directions. *Canadian Journal of Psychiatry. Revue Canadienne De Psychiatrie*.

[42] Snyder HR, Miyake A, Hankin BL (2015). Advancing understanding of executive function impairments and psychopathology: bridging the gap between clinical and cognitive approaches. *Frontiers in Psychology*.

[43] King MJ, MacDougall AG, Ferris SM, Levine B, MacQueen GM, McKinnon MC (2010). A review of factors that moderate autobiographical memory performance in patients with major depressive disorder. *Journal of Clinical and Experimental Neuropsychology*.

[44] Van Vreeswijk MF, De Wilde EJ (2004). Autobiographical memory specificity, psychopathology, depressed mood and the use of the Autobiographical Memory Test: a meta-analysis. *Behaviour Research and Therapy*.

[45] Bora E, Harrison BJ, Yücel M, Pantelis C (2013). Cognitive impairment in euthymic major depressive disorder: a meta-analysis. *Psychological Medicine*.

[46] Hasselbalch BJ, Knorr U, Kessing LV (2011). Cognitive impairment in the remitted state of unipolar depressive disorder: a systematic review. *Journal of Affective Disorders*.

[47] Nandrino JL, Pezard L, Posté A, Réveillère C, Beaune D (2002). Autobiographical memory in major depression: a comparison between first-episode and recurrent patients. *Psychopathology*.

[48] Alper J, Feng R, Verma G, Rutter S, Huang KH, Xie L (2023). Stress-related reduction of hippocampal subfield volumes in major depressive disorder: A 7-Tesla study. *Frontiers in Psychiatry*.

[49] Brosch K, Stein F, Schmitt S, Pfarr JK, Ringwald KG, Thomas-Odenthal F (2022). Reduced hippocampal gray matter volume is a common feature of patients with major depression, bipolar disorder, and schizophrenia spectrum disorders. *Molecular Psychiatry*.

[50] Nuninga JO, Mandl RCW, Boks MP, Bakker S, Somers M, Heringa SM (2020). Volume increase in the dentate gyrus after electroconvulsive therapy in depressed patients as measured with 7T. *Molecular Psychiatry*.

[51] Benjamin MJ, Cifelli A, Garrard P, Caine D, Jones FW (2015). The role of working memory and verbal fluency in autobiographical memory in early Alzheimer’s disease and matched controls. *Neuropsychologia*.

[52] Raes F, Hermans D, Williams JMG, Demyttenaere K, Sabbe B, Pieters G (2006). Is overgeneral autobiographical memory an isolated memory phenomenon in major depression?. *Memory (Hove, England)*.

[53] Sheldon S, Moscovitch M (2012). The nature and time-course of medial temporal lobe contributions to semantic retrieval: an fMRI study on verbal fluency. *Hippocampus*.

[54] Sumner JA, Griffith JW, Mineka S (2011). Examining the mechanisms of overgeneral autobiographical memory: capture and rumination, and impaired executive control. *Memory (Hove, England)*.

[55] Goldberg Z, Kuslak B, Kurtz MM (2020). A meta-analytic investigation of cognitive remediation for mood disorders: Efficacy and the role of study quality, sample and treatment factors. *Journal of Affective Disorders*.

